# Overexpression of B7-H3 correlates with aggressive clinicopathological characteristics in non-small cell lung cancer

**DOI:** 10.18632/oncotarget.13177

**Published:** 2016-11-07

**Authors:** Shanshan Wu, Xiangfei Zhao, Sudong Wu, Rui Du, Qi Zhu, Henghu Fang, Xinhong Zhang, Chunyang Zhang, Wei Zheng, Jihua Yang, Huasong Feng

**Affiliations:** ^1^ Postgraduate Team, Chinese PLA General Hospital, Beijing 100853, China; ^2^ Department of Radiation Oncology, Navy General Hospital, Beijing 100048, China

**Keywords:** meta-analysis, B7-H3, prognosis, lung cancer, clinical

## Abstract

Previous studies have investigated the prognostic significance of B7 homolog 3 (B7-H3) in non-small cell lung cancer (NSCLC), however, the results remain controversial. This study was aimed to determine the correlation between B7-H3 and survival as well as clnicalpathological characteristics in NSCLC using meta-analysis. We searched the electronic databases of PubMed, Embase, Web of Science, and China National Knowledge Infrastructure (CNKI) for relevant studies up to October 9, 2016. Pooled hazard ratios (HRs) and 95% confidence intervals (CIs) were used to estimate the impact of B7-H3 on overall survival (OS). Combined odds ratios (ORs) and 95%CIs were utilized to evaluate the correlations between B7-H3 and clinicalpathological features. This meta-analysis finally included 7 studies with 864 patients. The results showed that B7-H3 had no significant association with OS (HR=0.88, 95%CI: 0.36-2.13, p=0.776). High B7-H3 expression was a significant indicator of lymph node metastasis (OR=3.92, 95%CI: 2.65-5.81, p<0.001), and advanced TNM stage (OR=3.53, 95%CI: 2.45-5.09, p<0.001). B7-H3 has the potential to serve as a marker of tumor aggressiveness and lymph node metastasis in NSCLC. However, due to several limitations, further large-scale studies are needed to validate our results.

## INTRODUCTION

Lung cancer is the leading cause of cancer-related deaths worldwide [1]. Lung cancer consists of two main types: small cell lung cancer (SCLC) and non-small cell lung cancer (NSCLC). NSCLC accounts for approximately 85% of all lung cancer cases [2]. In recent decades, major advances have been achieved in surgical techniques, chemotherapy, radiotherapy, and immunotherapy for NSCLC. Unfortunately, treatment outcomes for NSCLC remain poor, with a 5-year survival rate being 15% [3]. Recent evidence suggests that several mechanisms involving tumor microenvironment results in immune defects in NSCLC, which is responsible for poor prognosis [4, 5]. Accordingly, there is a need to define immune-related molecular targets and mechanisms to stratify high risk individuals.

B7 homolog 3 (B7-H3) is a member of the co-inhibitory B7 family. B7-H3 was first cloned and named in 2001 and was reported to participate in the regulation of T cell mediated immune responses [6]. B7-H3 is broadly expressed at low levels in normal tissues [7]. Accumulating studies revealed that B7-H3 could inhibit T-cell proliferation, reduce production of cytokines, and suppress activation of transcription factors [8, 9]. Recent studies have shown that B7-H3 is upregulated in various malignant tumors including pancreatic cancer [10], prostate cancer [11, 12], renal cell carcinoma [13, 14], and NSCLC [15]. Nevertheless, the clinical significance and prognostic value of B7-H3 in NSCLC are controversial according to present studies [16-19]. In this setting, we searched the relevant studies and conducted this meta-analysis in order to gain a comprehensive understanding of the prognostic impact of B7-H3 on patients with NSCLC.

## RESULTS

### Study selection

**Figure 1:**
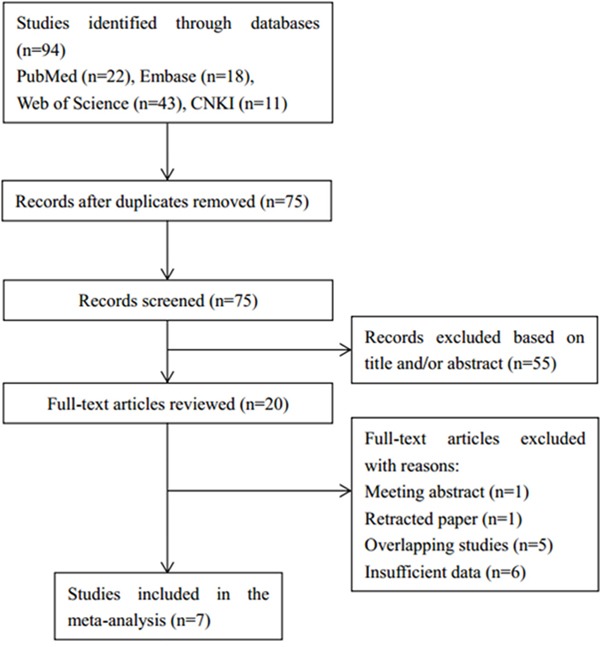
Flow diagram showing selection of studies

A flow chart describing the study selection process was shown in Figure 1. The initial search strategy identified a total of 94 studies. After duplicates were removed, 75 records were screened on the base of title and abstract. Among them, 20 articles were left for full-text evaluation. Afterwards, 13 of those 20 articles were discarded due to the following reasons: one was a meeting abstract, one was retracted, five were overlapping studies, and six were without sufficient data. At last, 7 studies [15, 19-24] were included for this meta-analysis.

### Study characteristics

The main characteristics of the 7 eligible studies were listed in Table 1. The studies were published from 2006 to 2016 and six studies were in English [15, 19-21, 23, 24] and one was in Chinese [22]. Six studies were from China [15, 19-23] and one study was from Japan [24]. The sample sizes ranged from 70 to 270 with a sum of 864. Four studies [19, 21, 23, 24] reported the correlation between B7-H3 and OS and all seven studies showed the association between B7-H3 and clinicalpathological features. Six studies [15, 19, 21-24] used immunohistochemistry (IHC) to detect B7-H3 expression and one [20] used enzyme linked immunosorbent assay (ELISA).

**Table 1:** General characteristics of the included studies

Study	Year	Country	Sample size	Gender (M/F)	TNM stage	Treatment	Research duration	Detection method	Positive (%)	Language
Sun	2006	China	70	49/21	I-III	Surgical resection	2003-2004	IHC	37.1	English
Zhang	2009	China	98	70/28	I-IV	Surgical resection	2004-2007	ELISA	48	English
Xu	2010	China	102	66/36	I-IV	Surgical resection	2006-2008	IHC	69.6	English
Feng	2015	China	86	41/45	I-IV	Surgical resection	2013-2014	IHC	47.7	Chinese
Jin	2015	China	110	83/27	I-III	Surgical resection	2006-2015	IHC	54.55	English
Mao	2015	China	128	91/37	I-III	Surgical resection	2005-2007	IHC	69.5	English
Inamura	2016	Japan	270	145/125	I-IV	Surgical resection	1995-2002	IHC	32	English

Abbreviations: IHC= immunohistochemistry; ELISA= enzyme linked immunosorbent assay.

### B7-H3 expression and overall survival

**Figure 2:**
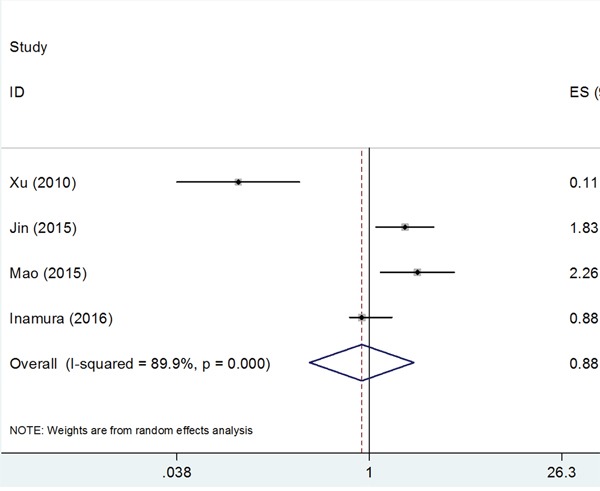
Forest plot depiction of the association between B7-H3 expression and overall survival in NSCLC

A total of 4 studies [19, 21, 23, 24] with 610 patients investigated the impact of B7-H3 on OS. Because of significant heterogeneity (*I*^2^=89.9%, P_h_<0.001), a random-effects model was utilized. The pooled HR and 95%CI were: HR=0.88, 95%CI: 0.36-2.13, p=0.776; Figure 2. The results suggested that there was no significant association between B7-H3 expression and OS in NSCLC.

### B7-H3 expression and clinicopothological characteristics

Relevant data were calculated to estimate the correlation between B7-H3 and 8 clinicalpathological parameters. These features included age, gender, lymph node metastasis, tumor differentiation, T stage, histology, TNM stage, and smoking history. The overall results were demonstrated in Table 2. The synthesized data showed that there were statistically significant connection between B7-H3 and lymph node metastasis (OR=3.92, 95%CI: 2.65-5.81, p<0.001), and TNM stage (OR=3.53, 95%CI: 2.45-5.09, p<0.001). However, the combined data revealed that there was no significant correlation between B7-H3 and age (OR=1, 95%CI: 0.75-1.33, p=0.991), gender (OR=1.46, 95%CI: 0.93-2.29, p=0.099), differentiation (OR=1.76, 95%CI: 0.74-4.15, p=0.199), T stage (OR=1.42, 95%CI: 0.73-2.75, p=0.303), histology (OR=0.73, 95%CI: 0.33-1.61, p=0.435), or smoking history (OR=1.22, 95%CI: 0.87-1.71, p=0.248).

**Table 2:** Association between B7-H3 and clinical parameters in NSCLC

Parameters	No. of studies	Effects model	OR (95%CI)	P-value	Heterogeneity	
					*I*^2^(%)	P_h_
Age	7	Fixed	1(0.75-1.33)	0.991	46.6	0.081
Gender	7	Random	1.46(0.93-2.29)	0.099	51.4	0.055
Lymph node metastasis	6	Fixed	3.92(2.65-5.81)	<0.001	43.5	0.116
Differentiation	6	Random	1.76(0.74-4.15)	0.199	79.2	<0.001
T stage	5	Random	1.42(0.73-2.75)	0.303	62.1	0.032
Histology	5	Random	0.73(0.33-1.61)	0.435	76.6	0.002
TNM stage	5	Fixed	3.53(2.45-5.09)	<0.001	0	0.592
Smoking history	5	Fixed	1.22(0.87-1.71)	0.248	0	0.88

P-value was for OR (95%CI). P_h_=P_heterogeneity_, P_h_ was for heterogeneity test.

### Publication bias

**Figure 3:**
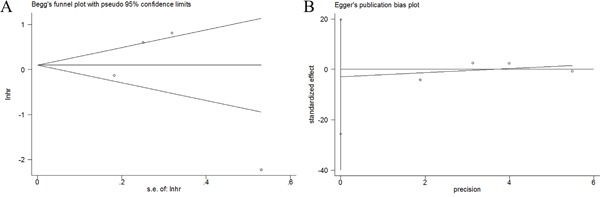
Publication bias detected by Begg's test and Egger's test **A.** Begg's test for OS. **B.** Egger's test for OS.

Potential publication bias statistics were determined by using both Begg's test and Egger's test. As shown in Figure 3, p-values for Begg's test and Egger's test were more than 0.05 (Begg's p=1 and Egger's p= 0.634 for OS). Therefore, no obvious publication bias was detected in our meta-analysis.

## DISCUSSION

NSCLC is one of the most devastating neoplasms around the world. The prognosis of NSCLC was not substantially improved despite therapeutic advancement. Recent progresses in tumor immunology identified a series of costimulatory molecules such as B7 homolog 1 (B7-H1, PD-L1, CD80), B7-H2 (CD 86), B7-H3 (CD 276), and B7-H4 (B7x, B7S1). These costimulatory molecules can combine with their receptors to mediate the intensity of immune responses in physiological and pathological conditions. Immunotherapy targeting B7-H1 has shown promising effects in advanced tumor patients including NSCLC [25, 26]. These findings encourage us to investigate the prognostic significance of B7-H3 in NSCLC through meta-analysis. In this study, 7 articles with 864 patients were included. The results illustrated that high B7-H3 expression was associated with presence of lymph node metastasis, and advanced TNM stage, whereas B7-H3 had no significant relationship with OS or other clinical characteristics. Our results suggested that B7-H3 was a potential stimulator for tumor cells dissemination and invasion. To the best of our knowledge, this is the first comprehensive meta-analysis to assess the prognostic role of B7-H3 in patients with NSCLC.

B7-H3 is a type I transmembrane protein which shares ~25% amino acid identity with B7-H1 and B7-H2 [6]. B7-H3 protein is widely expressed in peripheral tissues including osteoblasts, fibroblasts, human liver, bladder, placenta, and lymphoid organs [27]. Growing evidence showed that B7-H3 was implicated in inhibiting T cells-mediated immune reactions [28]. Furthermore, the interaction between CD4+CD25+ regulatory T cells (Tregs) and dendritic cells (DCs) induces expression of B7-H3 on DCs [29]. A number of studies demonstrated the prognostic effect of B7-H3 on different solid tumors. Yamato et al [10] reported that B7-H3 expression was significantly more intense in cases with lymph node metastasis and advanced pathological stage in pancreatic cancer. Yuan et al [30] found that B7-H3 overexpression could promote tumor cells migration and invasion in prostate cancer. Qin et al [14] showed that B7-H3 expression was associated with multiple adverse clinical and pathologic features in renal cell carcinoma. Moreover, Huang et al [31] revealed that B7-H3 levels were significantly associated with tumor size in patients with cervical cancer. Wang et al [32] disclosed that high B7-H3 expression was an indicator of advanced stage and common pulmonary metastasis in osteosarcoma. In the current meta-analysis, we found that B7-H3 overexpression was correlated with lymph node metastasis, and advanced TNM stage, which were in accordance with previous findings [10, 14, 31, 32]. Notably, some studies [12, 32] showed that patients with high tumor B7-H3 levels had shorter survival time and recurrence time. Interestingly, the data in our study did not suggest that B7-H3 expression predict poor survival in NSCLC. The reason may be that the total sample size in this meta-analysis is limited, which may introduce bias.

Despite its strengths, this meta-analysis still has several limitations. First, all primary studies included were from Asia, which may cause selection bias. Although many studies from other countries were searched, they were removed because they did not meet inclusion criteria. Second, the sample size was relatively small in this meta-analysis. More studies are especially needed to explore the relationship between B7-H3 and OS.

In conclusion, this meta-analysis illustrated that B7-H3 was significantly associated with lymph node metastasis, and advanced TNM stage in NSCLC. However, B7-H3 had no significant connection with OS in NSCLC. B7-H3 has the potential to serve as a marker of tumor aggressiveness and metastasis. Due to the limitations, further large-scale studies from different countries are warranted.

## MATERIALS AND METHODS

### Search strategy

This meta-analysis was performed referring to Preferred Reporting Items for Systematic Reviews and Meta-Analyses (PRISMA) guidelines [33]. Relevant studies were searched through the electronic platforms of PubMed, Embase, Web of Science, and China National Knowledge Infrastructure (CNKI) up to October 9, 2016. Search items included “B7-H3”, “B7H3”, “B7 homolog 3”, “CD276”, “lung cancer”, “lung carcinoma”, “non-small cell lung cancer”, and “NSCLC”. The reference lists were also manually searched to identify potentially related articles.

### Inclusion and exclusion criteria

Eligible studies were required to meet the following criteria: (1) the diagnosis of NSCLC was proven by pathological methods; (2) studies investigated the relationships between B7-H3 and overall survival (OS) or clinicopathological characteristics; (3) B7-H3 expression was measured by any method; (4) hazard ratio (HR) and 95% confidence interval (CI) of OS or odds ratio (OR) and 95%CI of clinicopathological characteristics were reported or could be calculated by Tierney's method [34]; (5) studies were published as full-text articles in English or Chinese; (6) if the same patient group was reported more than once, the most complete one was included. Animal studies, duplicate articles, abstracts and studies with insufficient information were removed from the analysis.

### Data extraction

All data were extracted by two independent investigators (SSW and XFZ) from eligible studies. Any disagreement between the two investigators was settled by discussion. The following information was extracted: first author, year of publication, study location, age, gender, sample size, TNM stage, treatment strategies, detection method for B7-H3, research duration, and publication language.

### Statistical analysis

HR with 95%CI was used to strength of association between B7-H3 and OS. Heterogeneity among studies was evaluated by using chi-square based Q-test [35] and Higgins *I*^2^ statistic [36]. Significant heterogeneity was defined as p for heterogeneity <0.10 or *I*^2^ >50%. If significant heterogeneity was found, a random-effects model was performed for analysis, otherwise, a fixed-effects model was applied. ORs and 95%CIs were utilized as the effect sizes to assess the association between B7-H3 and clinical parameters in NSCLC. Publication bias in meta-analysis was estimated using Begg's funnel plot and Egger's linear regression tests. P <0.05 was considered as statistically significant. All statistical analyses were accomplished using the STATA 12.0 software (Stata corp, College station, TX).
